# Effect of enamel-surface modifications on shear bond strength using different adhesive materials

**DOI:** 10.1186/s12903-022-02254-7

**Published:** 2022-06-07

**Authors:** Bo-wen Zheng, Shan Cao, Majedh Abdo Ali Al-Somairi, Jia He, Yi Liu

**Affiliations:** 1 Department of Orthodontics, School and Hospital of Stomatology, Liaoning Provincial Key Laboratory of Oral Diseases, China Medical University, Shenyang, 110002 China; 2 Department of Orthodontics and Dentofacial Orthopedics, Faculty of Dentistry, Ibb University, Ibb, Republic of Yemen

**Keywords:** Ceramic brackets, Surface treatments, Resin-modified glass ionomer cement, Shear bond strength

## Abstract

**Background:**

This study aimed to investigate the effect of enamel-surface modifications on the shear bond strength between ceramic brackets bonded using different adhesive materials and the enamel surface and to identify the most suitable clinical adhesive and bonding method. Whether the non-acid-etching treatment met the clinical bond strength was also determined.

**Methods:**

A total of 108 extracted premolars were divided into nine groups (n = 12) based on the different enamel-surface modification techniques (acid etching, deproteinization, and wetting). Group 1 was bonded with Transbond™ XT adhesive, whereas groups 2–9 were bonded with resin-modified glass ionomer cement (RMGIC). The treatment methods for each group were as follows: groups 1 and 2, acid etching; group 3, acid etching and wetting; group 4, acid etching and deproteinization; group 5, acid etching, deproteinization, and wetting; group 6, deproteinization; group 7, deproteinization and wetting; group 8, without treatment; and group 9, wetting. The samples' shear bond strength was measured using an universal testing machine. Adhesive remnant index (ARI) was examined using a stereomicroscope. The enamel-surface morphology was observed with a scanning electron microscope. One-way ANOVA with Tukey’s post-hoc test and chi-square test were used for statistical analysis, and *p* < 0.05 and α = 0.05 were considered statistically significant.

**Results:**

The ARIs of groups 1–5 and 6–9 were statistically significant (*p* = 0.000). The enamel surface of groups 1–5 was demineralized, and only a tiny amount of protein remained in groups 7 and 8, whereas a thick layer of protein remained in groups 8 and 9.

**Conclusions:**

RMGIC adhesive did not damage the enamel surface and achieved the required clinical bond strength. The enamel surface was better treated with 5.25% sodium hypochlorite preferably under non-acid-etching conditions.

## Background

Composite resin was originally used as an orthodontic bracket bonding agent in the 1970s. The use of resin enabled accurate bracket positioning and improved orthodontic-treatment efficiency [1].

Wilson and Kent [2] initially introduced glass ionomer cement (GIC) in the oral field and then launched it in the orthodontic field. GIC bonds chemically to enamel and dentin through the reaction between calcium and carboxylic acids in the teeth and GIC, respectively. Moreover, GIC can release fluoride into the oral cavity and prevent caries. GIC has a specific adhesion to tooth tissues, but it has a lower bond strength with a higher falling rate after fixing orthodontic brackets [3, 4].To increase the bond strength with the caries-resistance function of orthodontic brackets, the bonding agent denoted as resin-modified (RMGIC) appeared at the end of the twentieth century [5].

Studies have shown that resin may improve the flexural strength of GIC, and the bond strength of RMGIC can meet clinical requirements. The RMGIC micro-tensile bond strength was 33.15 MPa, which is much higher than the GIC bond strength of approximately 2.4–5.5 MPa [6, 7]. RMGIC can also release fluoride, has a certain degree of caries resistance, and is almost unaffected by enamel-surface dryness [7]. A bonding-strength range of 5.9–7.8 MPa is generally considered suitable for most orthodontic needs [1]. The falling rates of RMGIC and composite resin are reportedly 5% and 8.3%, respectively, which is not statistically significant [8].

The bond strength of orthodontic brackets depends on the bonding agent and the treated-surface properties of the enamel and bracket. Phosphoric acid is often used for enamel etching, which enables the formation of micropores on the enamel surface. Accordingly, adhesives can be infiltrated and the mechanical bonding is adequate [9].

Notably, certain factors may weaken or break the bond between the enamel surface and brackets. The oral cavity is a complex environment. Saliva and blood contamination are considered the most common causes of bonding failure because adhesive penetration is highly obstructed. Thus, it is advantageous for bonding agents to bond under the condition that the enamel might be contaminated with water and acid-etching [10].

Meanwhile, sodium hypochlorite (NaClO) solution provides specific properties such as disinfection and cleaning of root canals from impurities and organic substances. Previous studies have shown that pretreatment of enamel surface with NaOCl can increase the degree of penetration of adhesives into the enamel [11, 12]. Owing to the deproteinization of the enamel surface by NaClO, suitable acid etching is required to achieve better bond strength between the bracket and enamel [13, 14].

Previous studies have reported the bond strength of brackets on different surfaces (e.g., bleached tooth surface restorations and temporary restorations) and the bond strength of different adhesives; however, the optimal adhesive and bonding conditions have not yet been reported [15–18]. In the present study, the enamel surface was treated using different techniques such as acid etching, deproteinization, and wetting to investigate the effect of enamel-surface modifications on the shear bond strength (SBS) between the ceramic brackets bonded to the enamel surface used in RMGIC to identify the most suitable clinical adhesive and bonding method.

## Methods

### Specimens and surface treatments

The study was approved by the ethics committee of the Stomatology Hospital of China Medical University, Liaoning, China (no. CMUKQ-2021-024). Written informed consent was obtained from all participating adults and parents. All procedures were performed in accordance with the principles of the declaration of Helsinki.

A total of 108 sound extracted human premolar teeth were selected for surface modification. The teeth were cleaned with a slurry of fluoride-free pumice paste (Nada Pumice Paste; Preventech, Matthews, NC, USA) and preserved in a 0.1% musk-grass phenol solution until use. Specific inclusion criteria were considered for the extracted teeth, such as teeth that were entirely developed and normal, with no caries or cracks on all surfaces. The selected teeth were not associated with deposited pigments or calculus. Additionally, they were not subjected to surface pretreatment with chemical reagents such as alcohol, formalin, or hydrogen peroxide. The specimens were randomly assigned to nine groups (n = 12) treated with different enamel-surface modifications (acid etching, deproteinization, and wetting). The sample size was predetermined as 12 specimens per group by augmenting the sample sizes used in a previous study [19]. The experimental groupings are presented in Table 1. Group 1 was bonded with Transbond™ XT adhesive (3 M™ Unitek Transbond™ XT CA, USA), whereas groups 2–9 were bonded with RMGIC (GC Fuji ORTHO™ LC, USA). In a typical procedure of acid etching, the enamel surface was etched with 35% phosphoric acid (BISCO, IL, USA) for 30 s, rinsed for 30 s, and air dried for 30 s. In a typical procedure of deproteinization, 5.25% NaClO was applied on the tooth surface with slight brushing for 1 min, rinsed for 30 s, and gently air dried for 30 s. The detailed process of wetting the enamel surface was as follows: it was wiped with a half-dry cotton roll moistened with distilled water, squeezed until no water drops remained, and kept under moist conditions. After bonding the brackets, the specimens were immersed in a water bath for 24 h at room temperature.

**Table 1:** Experimental grouping and shear bond strength (MPa) of each group

Group	NaClO	Acid etchant	Moisture	Bonding agent	Shear bond strength Mean ± SD
1	−	+	−	Transbond™ XT	29.19 ± 6.01
2	−	+	−	GC Fuji LC	33.49 ± 6.71
3	−	+	+	GC Fuji LC	22.62 ± 4.73
4	+	+	−	GC Fuji LC	30.96 ± 2.96
5	+	+	+	GC Fuji LC	28.09 ± 3.84
6	+	−	−	GC Fuji LC	13.44 ± 2.14
7	+	−	+	GC Fuji LC	12.67 ± 2.80
8	−	−	−	GC Fuji LC	6.50 ± 1.66
9	−	−	+	GC Fuji LC	7.44 ± 2.54

### Bracket-bonding method

Ceramic brackets without any surface procedures were positioned and pressed in the center of the clinical crown on the buccal surface of the isolated tooth by using bracket-holding forceps (3 M™ Unitek, CA, USA). The excess adhesive around the bracket was then removed with a probe. A photosensitive lamp was used in all bonding procedures for 10 s per direction, including the medial, distal, occlusal, and gingival margins.

### SBS test

**Fig. 1:**
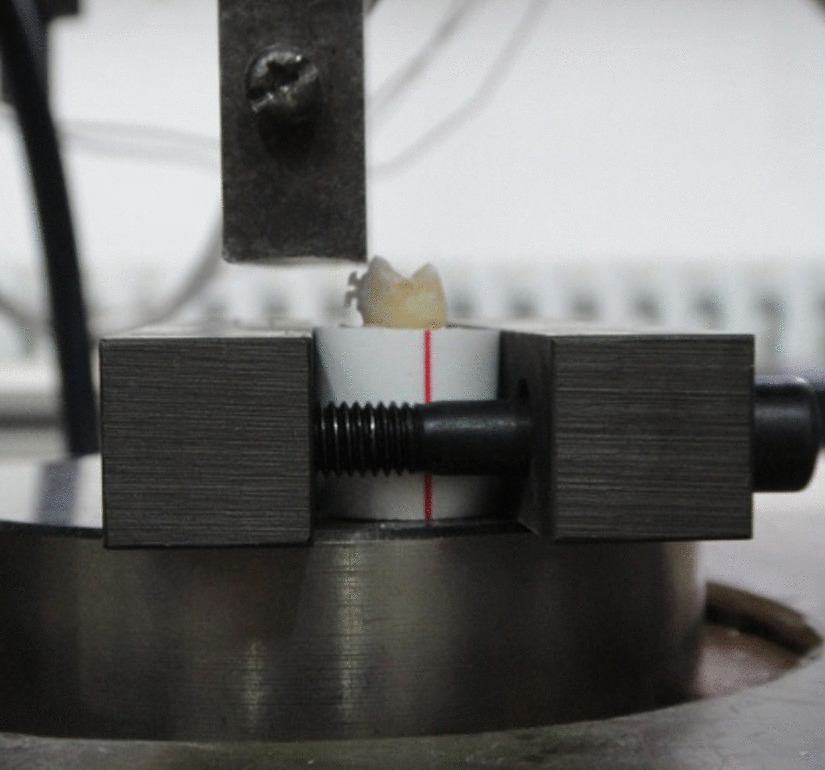
During performing shear bond strength testing between ceramic bracket and tooth surface

The SBS test was performed using an electronic universal testing machine (Instron Model 8874, UK). The specimen was fixed in a block of self-curing acrylic resin at a level of 1 mm below the cemento-enamel junction. The test was performed at a crosshead speed of 1 mm/min, as illustrated in Fig. 1. A load was applied until bonding failure occurred. The SBS test was recorded in Newtons and then converted into MPa using the following formula:

1 $${\text{SBS}} = \frac{P}{S}({\text{MPa}})$$

where *P* and *S* are the maximum load and bonding area, respectively.

### Adhesive remnant index (ARI) evaluation

After debonding the brackets from the enamel surface, the teeth were rinsed and gently air dried. ARIs were measured using a stereomicroscope with 10 × magnification (AmScope SE400-Z, Irvine, CA, USA) to observe the cross-sectional view of the specimens. The percentage of adhesive remaining on the enamel surface was measured according to the ARI, ranging from 0 to 3 [20]. The remnant of the tooth surface without adhesive was recorded as 0, the tooth surface with less than half of the adhesive was recorded as 1, the tooth surface with more than half percent of the adhesive was recorded as 2, and all adhesives that remained on the enamel tooth surface was recorded as 3.

### Scanning electron microscopy (SEM) analysis

SEM (ZEISS Goettingen, Germany) observation was conducted under vacuum conditions because of the difficulty in observing the enamel-surface morphology under moist conditions. Prior to bonding brackets, SEM was used to observe different surface treatments at 1000 × and 10,000 × magnification, respectively.

### Statistical analysis

The collected data were analyzed using SPSS software (17.0 version; IBM Corporation). One-way ANOVA and chi-square test with Tukey’s post-hoc test were used, and *p* < 0.05 and α = 0.05 were considered statistically significant.

## Results

The SBS for each group were shown in Tables 1 and 2, respectively The SBS values for each group were 29.19 ± 6.01, 33.49 ± 6.71, 22.62 ± 4.73, 30.96 ± 2.96, 28.09 ± 3.84, 13.44 ± 2.14, 12.67 ± 2.80, 6.50 ± 1.66, 7.44 ± 2.54 MPa respectively. The results of the ANOVA indicated a significant difference between groups (*P* < 0.05). The SBS values for various enamel-surface treatments significantly differed. The SBS of the acid-etching and NaClO treatment groups in the non-acid-etching groups were greater than the clinically required bond strength of 5.9–7.8 MPa. The SBS of the groups without acid etching and NaClO treatment was less close to the clinically required bond strength. Pairwise comparisons between the groups with the Tukey post hoc test shown in Table 2 revealed that each acid-etching group showed significantly higher SBS when compared to each non-acid etching group, each non-acid etching with NaClO treatment group showed significantly higher SBS than each non-acid etching without NaClO treatment group, and acid- etching with moisture group showed significantly lower SBS than acid etching without moisture group.

**Table 2:** Pairwise comparisons of the groups in terms of the shear bond strength (SBS)

P	1	2	3	4	5	6	7	8	9
1		0.946	0.188	1.000	1.000	0.000*	0.000*	0.000*	0.000*
2	0.946		0.006*	0.998	0.492	0.000*	0.000*	0.000*	0.000*
3	0.188	0.006*		0.052	0.145	0.001*	0.000*	0.000*	0.000*
4	1.000	0.998	0.052		0.743	0.000*	0.000*	0.000*	0.000*
5	1.000	0.492	0.145	0.743		0.000*	0.000*	0.000*	0.000*
6	0.000*	0.000*	0.001*	0.000*	0.000*		1.000	0.000*	0.000*
7	0.000*	0.000*	0.000*	0.000*	0.000*	1.000		0.000*	0.003*
8	0.000*	0.000*	0.000*	0.000*	0.000*	0.000*	0.000*		1.000
9	0.000*	0.000*	0.000*	0.000*	0.000*	0.000*	0.003*	1.000	

^*^p < 0.05 is statistically significant

### Adhesive remnant index

Table 3 displays the ARI after measuring the SBS. Table 4 presents the chi-square analysis results for the ARI. The differences between the acid-etched and non-acid-etched groups were statistically significant. In the acid-etching groups, a significant difference existed between the Transbond™ XT adhesive with NaClO and the moisture-modification group and the RMGIC adhesive without NaClO and the moisture-modification group, and no significant difference existed among the other groups. No significant difference existed between non-acid-etching groups.

**Table 3:** ARI scores

Group	0	1	2	3
1	0	0	4	8
2	0	3	6	3
3	0	3	6	3
4	0	3	3	6
5	0	7	3	2
6	9	3	0	0
7	9	3	0	0
8	12	0	0	0
9	12	0	0	0

**Table 4:** Chi-square test results of ARI

P	1	2	3	4	5	6	7	8	9
1		0.059	0.059	0.180	0.005*	0.000*	0.000*	0.000*	0.000*
2	0.059		1.000	0.368	0.247	0.000*	0.000*	0.000*	0.000*
3	0.059	1.000		0.368	0.247	0.000*	0.000*	0.000*	0.000*
4	0.180	0.368	0.368		0.165	0.000*	0.000*	0.000*	0.000*
5	0.005*	0.247	0.247	0.165		0.001*	0.001*	0.000*	0.000*
6	0.000*	0.000*	0.000*	0.000*	0.001*		1.000	0.064	0.064
7	0.000*	0.000*	0.000*	0.000*	0.000*	1.000		0.000*	0.003*
8	0.000*	0.000*	0.000*	0.000*	0.000*	0.064	0.064		1.000
9	0.000*	0.000*	0.000*	0.000*	0.000*	0.064	0.064	1.000	

*p < 0.05 is statistically significant

### Scanning electron microscopy

**Fig. 2:**
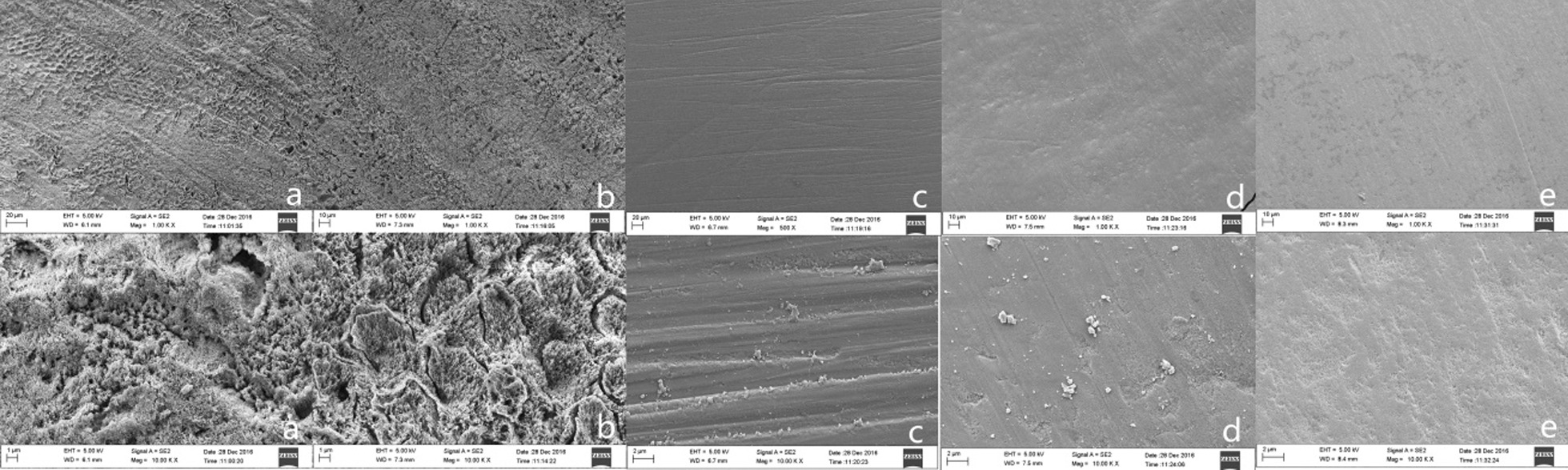
SEM results of enamel-surface treatment with different techniques: **a** 35% phosphoric acid etching of treated enamel surface, **b** 5.25% NaClO + 35% phosphoric acid etching of treated enamel surface, **c** 5.25% NaClO of the treated enamel surface, **d** only polished surface of the enamel surface, and **e** no treatment of the enamel surface

For bonding brackets, different enamel-surface treatments were performed. Then, SEM was conducted at 1000 × and 10,000 × magnification. SEM showed demineralized etched enamel surfaces with 35% phosphoric acid (Fig. 2a). The enamel surface treated with 5.25% NaClO and 35% phosphoric acid showed phosphoric acid dissolution in the enamel and peripheral areas (Fig. 2b). The enamel surface treated with 5.25% NaClO showed only a tiny amount of protein residue on the enamel surface, as shown in Fig. 2c. For only the polished surface of enamel surface, the SEM image showed a large amount of protein remnants on the enamel surface, and polished marks cannot be clearly observed, as shown in Fig. 2d. SEM shows a thick layer of protein for the last group, which did not undergo enamel-surface treatment, as shown in Fig. 2e.

## Discussion

Long-term fixed appliance treatment is prone to enamel demineralization and the formation of chalky white spots, resulting in caries with an incidence of 95% [21, 22]. The Transbond™ XT bonding system is the most extensively used bonding agent and is often used as the standard for evaluating bond strength with other bonding agents [6, 23]. Accordingly, the current study used Transbond™ XT as a control group to assess RMGIC (GC Fuji ORTHO™ LC, USA). Our results demonstrated that deproteinization of the enamel surface by using 5.25% NaClO can improve the bond strength of RMGIC to the enamel surface, meeting the clinical requirements (5.9–7.8 MPa) in the absence of acid etching [1]. Furthermore, moisture and saliva contamination reduced the bond strength of RMGIC, but its adhesive strength remained within the clinical requirements, and fluorine was released to prevent demineralization.

RMGIC is a hybrid of GIC and composite resins containing traditional glass ionomers, monomer components, and related initiator systems [24]. The curing mechanism of RMGIC involves the acid–base reaction of GIC and the free-radical polymerization of light-cured resins [10]. After mixing RMGIC powder, the acid–base reaction begins. When light curing was performed, the resin component immediately initiated polymerization. We confirmed that the shear bond and tensile strength test outcomes were more significant after 24 h than performing the test directly after 30 min [23].

Acid etching is the standard approach for enamel bonding in which hydroxyapatite crystals on the enamel surface are dissolved. It also increases the wettability and free energy the of enamel surface and is conducive to the adhesion between enamel and resin materials [7]. Before etching, the enamel is polished and cleaned. However, a large amount of protein remains on the enamel surface after polishing [25]. The degree of enamel demineralization after acid etching varies because of different degrees of mineralization on the enamel surface. Phosphoric acid reportedly cannot etch the enamel surface thoroughly. Only 2% exhibits ideal acid etching [25, 26].

Furthermore, NaClO has an antibacterial effect that dissolves organic material without damaging healthy tissue or tooth structure [13]. The enamel surface can be deproteinized with NaClO before bonding to increase the penetration of the adhesive into the enamel and further improve the bond strength. Thus, prior to acid etching, 5.25% NaClO is applied onto the enamel surface for 1 min to improve the etching property [11, 12]. The pH of NaClO is similar to that of calcium hydroxide, which reacts with OH– to form NaOH and HClO. NaOH and fatty acids react to reduce the surface tension and neutralize neutralizing the amino acids through HClO acid etching. Cl– action and cell metabolism permit OH– to bind to Ca^2+^ to form Ca(OH)2 [27]. The reaction of NaClO with the soft tissue is mild and presents a chlorinated odor.

In the current study, acid-etched and non-acid-etched groups showed statistically significant differences in bonding-strength measurements. The bond strengths of the formed were significantly higher than those of the latter. This finding may be due to the fact that the bonding between the RMGIC and the treated enamel surface achieved better strength through acid etching and adequate mechanical retention between the resin materials and the micropores of the etched enamel. RMGIC without acid etching treatment of the enamel bonding relied only on the retention of the glass-ionomer chemical reaction. Results showed that in the acid-etching groups, no statistically significant difference in bond strength existed between the Transbond™ XT adhesive group and RMGIC groups. Our results were similar to those reported by Cheng et al. [9]. They found that the bond strength of RMGIC to enamel after acid etching was greater than that of the composite resin. However, the current results were inconsistent with those of Yassaei et al. [26]. They treated enamel surface with 37% phosphoric acid and 10% polyacrylic acid by using a composite resin and RMGIC for bonding with ceramic brackets. They found that the bond strength of the composite resin is significantly greater than that of RMGIC.

In the etching groups, deproteinization with 5.25% NaClO and acid etching of the surface under the same conditions as the experimental group with a dry surface showed greater bond strength than in the group with a wet surface. In the acid-etching groups, the moisture and non-moisture groups had statistical significance, in which the bond strength of the former was lower than that of the latter. This finding was due to the acid etching deproteinizing the enamel surface. Under moist conditions, the pores formed on the enamel surface were clogged. The bond strength of the experimental groups treated with 5.25% NaClO before acid etching did not significantly differ from that of the control group. Treatment of the enamel surface with 5.25% NaClO prior to acid etching removed organic constituents, such as proteins from the enamel surface. Consequently, the surface was uniformly etched, consistent with the findings of Ayman et al. [28]. Previous studies have shown that the bond strength of an agent increased.

Notably, the difference was not statistically significant after 5.25% NaClO enamel-surface treatment [14]. However, Justus et al. [29] showed that the bond strength between RMGIC and the composite resin significantly increased after treating the enamel surface with 5.25% NaClO. This finding may be due to the use of 10% acid as a bonding agent in the former, whereas the latter used 37% phosphoric acid. Polyacrylic acid (10%) did not affect the deep areas and inflicted less damage to the enamel surface, so the enamel bond strength was low.

In the non-etching group, bond strength was significantly reduced, but the results were within the required clinical bond strength of 5.9–7.8 MPa. Moreover, the bond strength of the 5.25% NaClO-treated experimental group was significantly higher than that of the experimental group without 5.25% NaClO. This result indicated that 5.25% NaClO can deproteinize and expose the entire enamel surface. RMGIC can perform the acid–base reaction inside the glass ion and fully bond with the enamel surface under the unetched condition to achieve the clinically required bond strength. No significant difference in bond strength existed between the moisture and non-moisture groups, indicating that the surface treatment with NaClO was unaffected even in a humid environment.

The bonding failure of the enamel-treated surface with acid etching occurred primarily at the adhesive–bracket interface. Surface fracture of the enamel without acid etching occurred primarily on the enamel–adhesive surface. The enamel surface remained a high-bond-strength agent at the adhesive–bracket interface after the brackets fell off. This phenomenon may contribute to the formation of sufficient bond strength between the adhesive and enamel surface, which remained intact. ARI is one of the standard auxiliary indices used to evaluate the bonding properties of adhesives and can better respond to the failure position of the bonding agent. Our study showed that enamel thickness was reduced by the process of polishing, etching, bonding, and removal of residual bonding agents during orthodontic treatment at approximately 125 µm [30]. Therefore, for orthodontic brackets, selecting the appropriate bonding agent system was vital to keep the enamel surface intact after bracket removal.

In the current study, most of the interface failures of acid-etching groups were at the bonding agent–bracket interface. Conversely, Yassaei et al. [26] showed that the bonding failure of RMGIC occurs at the enamel–adhesive interface. The interface failure of non-acid-etching groups primarily occurs at the enamel–adhesive interface, indicating that the RMGIC relies mostly on chemical retention by the acid–alkali reaction inside the glass ions on enamel surfaces that are not acid etched. Other studies have suggested that enamel may fracture when subjected to SBS, particularly when applying a 5 MPa load [31]. Herein, the enamel surface of the non-acid-etching group was treated with 5.25% NaClO. The bond strength measured in the non-moisture group was 13.44 ± 2.14 MPa, and that measured in the moisture group was 12.67 ± 2.80 MPa. These results met the clinical requirements of bond strength and did not inflict damage to the enamel surface. Recent studies have also shown that resin-modified glass ionomer adhesives can inhibit the growth of *Streptococcus* mutans and reduce demineralization [32, 33].

SEM images showed that the enamel surface treated with 35% phosphoric acid underwent enamel-center demineralization and phosphoacrylate dissolution, but the interstitial and peripheral enamel parts were intact. Using 5.25% NaClO and 35% phosphoric acid, demineralized enamel-glazed parts were observed. Phosphoacrylate was also used to dilute the peripheral area of the glazed part. The methods used for retention purposes were based on previously described techniques. The porous area provided adequate retention over a wider surface area, depending on the size and depth of the pores. Acid etching did not result in a clear deep porous morphology and absence of micromechanical retention [34]. To observe the protein coverage of the enamel surface, the three groups were polished with 5.25% NaClO without any further treatments. Results showed that 5.25% NaClO had an acceptable deproteinization effect. The removal of the proteins by chemical means had no major effect on the elastic modulus and hardness of the dental enamel [35], as well as the laser surface treatments on the SBS between zirconia and veneering ceramic [36, 37]. In recent years, studies have been conducted on the application of CO_2_ laser pretreatment of the enamel surface to prevent enamel demineralization owing to acid erosion. However, these methods are complex and unstable [38, 39]. Therefore, enamel-surface treatment with 5.25% NaClO before bonding the bracket to the enamel by using RMGIC may provide good bond strength and inhibit enamel demineralization.

Recently, patients have requested the use of ceramic brackets because of their demand for maintaining an aesthetic smile. Ceramic brackets are hard, and their bond strength is extremely high. However, despite the hard properties of ceramic materials, they exhibit brittleness. Thus, changes in the ceramic bracket size of approximately 1% may affect their fracture and bond strength [38]. Bracket fractures reportedly lead to a large amount of bracket material being retained on the tooth surface, leading to a high risk of enamel damage during grinding [23, 38]. Hence, ceramic brackets may require moderate bond strength with the enamel surface rather than strong bonding to minimize the risk of enamel damage.

## Conclusions

In the case of enamel etching, the bond strength of RMGIC was comparable to that of the composite resin. In the absence of acid etching, the bond strength of RMGIC to the enamel surface met clinical requirements. Furthermore, moist and saliva contamination reduced the bond strength of RMGIC, but its adhesive strength was still within clinical requirements. Furthermore, the deproteinization of enamel surface using 5.25% NaClO can improve the bond strength of resin material. Finally, we recommended treating the enamel surface with 5.25% NaClO and bonding ceramic brackets with RMGIC onto the enamel surface.

## Data Availability

The datasets generated and analyzed in this study are not publicly available because of (ownership of data) but are available from the corresponding author upon reasonable request. All data and materials are available at the Stomatology Hospital, China Medical University, Liaoning, China.

## Abbreviations

RMGIC: Resin modified glass ionomer cement
GIC: Glass ionomer cement
SBS: Shear bond strength
ARI: Adhesive remnant index
SEM: Scanning electron microscopy
NaClO: Sodium hypochlorite
